# Topical retinoids: Novel derivatives, nano lipid‐based carriers, and combinations to improve chemical instability and skin irritation

**DOI:** 10.1111/jocd.16415

**Published:** 2024-07-01

**Authors:** Jiangming Zhong, Nan Zhao, Qingle Song, Zhiyun Du, Peng Shu

**Affiliations:** ^1^ HBN Research Institute and Biological Laboratory Shenzhen Hujia Technology Co., Ltd. Shenzhen Guangdong China; ^2^ State Key Laboratory Basis of Xinjiang Indigenous Medicinal Plants Resource Utilization, CAS Key Laboratory of Chemistry of Plant Resources in Arid Regions, Xinjiang Technical Institute of Physics and Chemistry Chinese Academy of Sciences Beijing China; ^3^ University of Chinese Academy of Sciences Beijing China; ^4^ School of Biomedical and Pharmaceutical Sciences Guangdong University of Technology Guangzhou Guangdong China

**Keywords:** chemical instability, nano lipid‐based carriers, retinoid derivatives, retinoids, skin irritation

## Abstract

**Background:**

Retinoids, defined as synthetic or natural derivatives of vitamin A, have been extensively studied as anti‐aging molecules that are widely applied in cosmetics. However, due to their physicochemical property, retinoids are highly unstable and extremely sensitive to light, oxygen, and temperature. Moreover, topical application of retinoids often leads to cutaneous irritation. These instabilities and irritant properties of retinoids limit their application in cosmetic and pharmaceutical products.

**Aim:**

Our study aimed to provide a systematic review to summarize the mechanisms underlying the instability and irritant properties of retinoids, as well as recent developments in addressing these challenges.

**Methods:**

A comprehensive PubMed search was conducted using the following keywords: retinoids, chemical instability, skin irritation, retinoid derivatives, nano lipid‐based carriers, liposomes, penetration‐enhancer vesicles, ethosomes, niosomes, nanoemulsions, solid lipid nanoparticles, vitamins, soothing and hydrating agents, antioxidants and metal chelator and retinol combinations. Relevant researches published between 1968 and 2023 and studies related to these reports were reviewed.

**Results:**

The development of new retinoid derivatives, the utilization of new delivery systems like nano lipid‐based carriers and the combination with other compounds like vitamins, soothing agents, antioxidants and metal chelator have been explored to improve the stability, bioavailability, and toxicity of the retinoid family.

**Conclusions:**

Through advancements in formulation techniques, structure modification of retinoid derivatives and development of novel nano lipid‐based carriers, the chemical instability and skin irritation of retinoids has been mitigated, ensuring their efficacy and potency over extended periods.

## 1 INTRODUCTION

Retinoids are an umbrella term for entire family of natural or synthetic vitamin A derivatives, and they usually have a head‐to‐tail structure containing four isoprene units.^1^ The term “retinoids” is used to describe compounds that consist of a beta‐ionone ring and a polyene side chain with a specific functional group. These functional groups can include an alcohol group, which results in retinol; an al‐dehyde group, leading to retinal; an acid group, producing retinoic acid (RA); or an ester group, resulting in retinyl ester. Retinol and retinyl ester are types of compounds that are commonly referred to as vitamin A and can be obtained through our diet. However, in order to be biologically active, retinol is converted to retinaldehyde followed by RA by dehydrogenases in a two‐step oxidation reaction.^2^, ^3^ RA exists in different forms, with the two most prevalent forms being all‐trans RA (ATRA) and 9‐cis RA.^4^ RA itself is rapidly metabolized by cytochrome P450 (CYPs), resulting the hydroxylation of ATRA and inactivation of its functions.^4^

Based on their molecular and structural properties, retinoids can be divided into three generations. First‐generation retinoids, such as retinol and its metabolites, are either naturally occurring or non‐aromatic compounds modified from the polyene vitamin. They do not act selectively towards retinoid receptors. Second‐generation retinoids are synthetic compounds that are either monoaromatic retinoids, where the cyclohexene ring is replaced by a benzene ring, or synthetic analogues of vitamin A. Third‐generation retinoids with cyclic polyene side‐chain are recognized by selective activity towards RA receptors‐β (RAR‐β) and RA receptors‐γ (RAR‐γ) receptors.^1^, ^5^, ^6^ The newly developed fourth‐generation of retinoids are characterized by binding selectively to RAR‐γ pre‐dominantly in epidermis.^7^

Retinoids play important roles in regulating a wide range of biological processes, including cell proliferation, differentiation, apoptosis, inflammation and development.^8^ Therefore, they have also been demonstrated in clinical pharmacotherapy for the treatment of vision‐related disorders, skin‐related disorders, and cancer.^8^, ^9^, ^10^ Retinol has also been widely used in cosmetic formulations to reduce wrinkles and improve cellulite and has been approved by the Food and Drug Administration (FDA) for anti‐aging treatments since 1996.^11^ The anti‐aging and anti‐photoaging effects^12^ of retinoids are based on the promotion of keratinocyte proliferation^13^, ^14^ collagen and elastin fiber synthesis,^15^ and the inhibition of collagen degradation by inhibiting metalloproteinase activity.^16^, ^17^ These effects ultimately result in a deceleration of the aging process, enhancement of repair mechanisms, and reduction of wrinkles and hyperpigmentation.

Nevertheless, the vast cosmetic and clinical applications of retinoids have been limited by three main obstacles. First, the poorly water‐soluble retinoids make them incorporative in a suitable aqueous vehicle, in which the water solubility of retinaldehyde, retinol and RA are about 0.1 μM at room temperature in pH 7.3.^18^, ^19^ Second, retinoids are photosensitive especially towards Ultraviolet A (UVA) lights and the photodamage is amplified by oxygen and aeration.^20^ Lastly, retinoids induce various irritating side effects during topical application. These include severe local irritation in the form of a burning sensation, itching, erythema, peeling, or dryness.^21^ These properties reduce the bioavailability of retinoids, thereby limiting their therapeutic efficacy. Therefore, researchers have developed several synthetic retinoid derivatives with improved stability and thus their utilization efficiency.

In this review, we try to summarize the crucial factors of instability and irritation of retinoids and provide a synopsis of the main updated advances in solving these obstacles, benefiting future retinoid use and research.

### 1.1 Chemical instability of retinoids

Retinoids consist of a cyclic six‐carbon ring attached to a polyene hydrophobic tail containing four isoprene units. The oxidation of the alcohol end of retinol results in aldehyde formation (all‐trans retinaldehyde or retinal), which is further oxidized to carboxylic acid (all‐trans RA or tretinoin). Moreover, unsaturated conjugated double bonds make them susceptible towards oxidation under the influence of various environmental and chemical factors including lights, temperature, strong acids and oxygen or peroxides.^22^ Thereby the presence of even trace amounts of acid in anhydrous solvent systems can lead to changes in the molecular structure of retinoids.^23^

The degradation of retinoids involves reactions such as isomerization, molecular fragmentation and chemical oxidation. For example, the double bonds present in the polyene chain of retinoids can undergo cis‐trans isomerization.^24^ In standpoint of the stability, retinyl palmitate is more heat‐resistant compared to retinol and therefore is used as the preferred retinoid for dermatological formulations. A recent comprehensive study adopting high‐performance liquid chromatography method with UV detection (UPLC‐UV) methodology was conducted to study the stability and degermation kinetics in most used cosmetic retinoids (retinol, retinyl pal‐mitate, β‐carotene and hydroxypinacolone retinoate (HPR)).^25^ The instability of retinoids was observed in nearly all products, with a decline ranging from 0% to 80% after 6 months at 25°C and more pounced decline ranging from 40% to 100% under 40°C. Light degradation was more significant than temperature‐induced degradation and the stability of the HPR was the most prominent among the others.^25^ Nevertheless, the instability of retinoids varied depending on the formulation of the cosmetics and different concentrations.

### 1.2 Skin irritation of topical retinoids

The use of topical retinoids can cause a variety of skin irritation including erythema, flaking, dryness, burning sensation and pruritus.^21^, ^26^ These symptoms are indicative of a specific type of irritant contact dermatitis known as “retinoid dermatitis”. Additionally, retinoids can directly enhance the excitability of nociceptors, causing epidermal keratinocytes, melanocytes and fibroblasts to secrete cytokines which further sensitize peripheral nociceptive fibers and lead to neurogenic inflammation and pain.^27^

The main contributors towards the retinoid‐induced skin irritation can be attributed to three categories (Figure.1). The first reason for retinoid‐induced irritation is a receptor‐mediated phenomenon.^28^ RA receptors (RAR), the natural ligands of RA have three isotypes (RAR‐α, ‐β, ‐γ), of which RAR‐γ that mainly ex‐pressed in skin tissues was characterized to induce skin irritation in rhino mouse and rabbit models.^28^ According to reports, increased RAR activity and a lack of specificity to particular subtypes in retinoids could also associated with side effects like skin irritation.^29^, ^30^ Retinoids are also specific towards transient receptor potential channel vanilloid subtype 1 (TRPV1) activators that excite injurious sensory neurons and evoke sensory hypersensitivity response.^21^ Therefore, selective RAR‐γ or TRPV1 antagonists may provide solution to lessen the irritation response.

**FIGURE 1:**
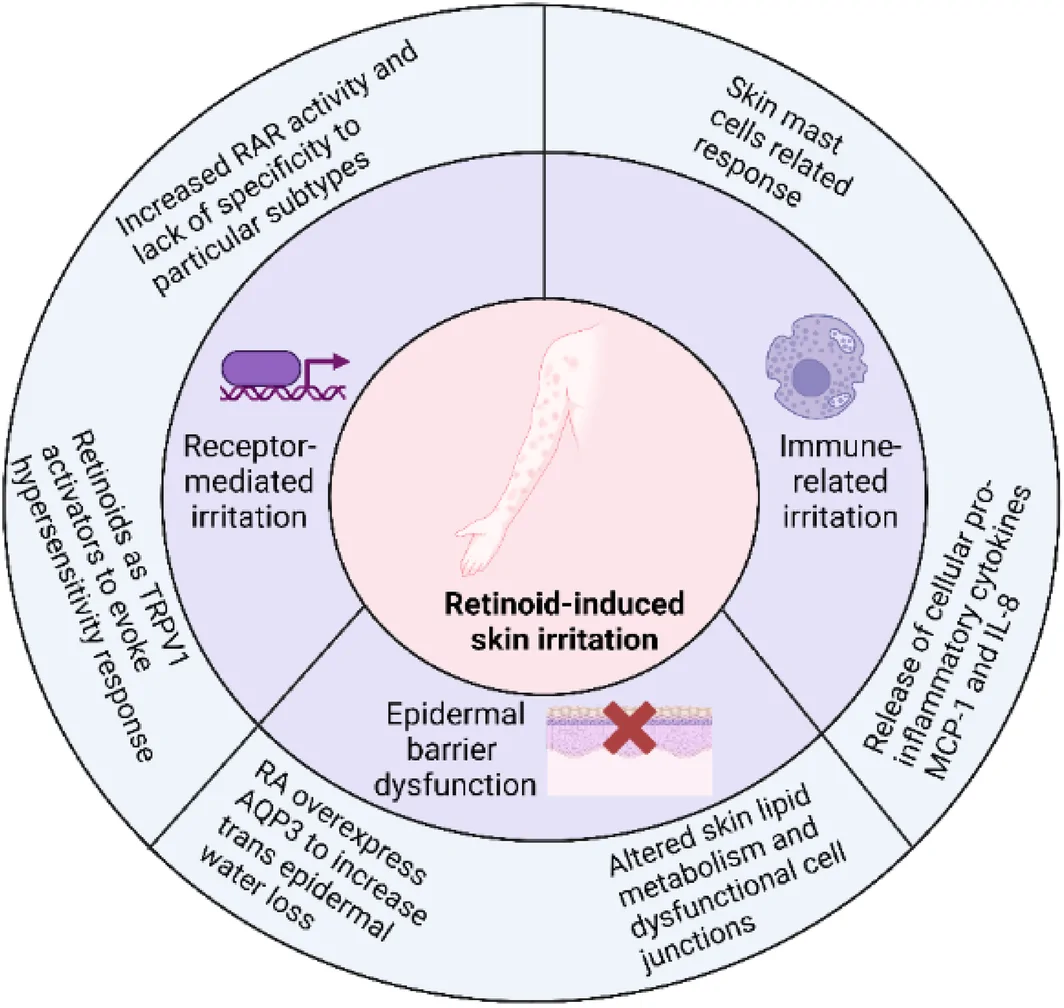
Schematic plot of the main causes for retinoids‐induced skin irritation.

Second, RA can elicit immune‐related functions. Skin mast cells (MCs) that originate from hematopoiesis and develop in skin, have been identified as the target for RA.^31^ Upon stimulation by RA, skin MCs increase the expression of pro‐inflammatory mediators, including interleukin‐1 beta (IL‐1β), interleukin‐8 (IL‐8), and tumor necrosis factor α (TNF‐α). The susceptibility of MCs towards RA depends on the abundance of RAR‐α expression, which is the receptor that regulates MC cytokine responses.^31^ At the same time, the damage caused by RA to the skin leads to the release of cellular inflammatory factors, among which the pro‐inflammatory cytokines monocyte chemoattractant protein‐1 (MCP‐1) and IL‐8 mainly mediate retinoids‐induced skin irritation.^26^, ^32^

Third, the retinoid‐induced skin irritation is associated with epidermal barrier dysfunction.^33^ Researchers found that treatment with RA showed significant epidermal hyperproliferation and hyperkeratosis, suggesting abnormal lipid droplets and intercellular junctions in the stratum corneum, indicating altered skin lipid metabolism and dysfunctional cell junctions.^33^ RA affects the expression of factors related to epidermal barrier function, including those associated with tight junctions (TJ), keratinized envelopes, lipids, proteases, protease inhibitors, and transcription factors.^34^ Claudin‐1 and ‐4 are proteins involved in TJ and have a key role in epidermal barrier function.^34^, ^35^ The data suggest that RA has a dual effect on epidermal barrier genes, as it down regulates claudin‐1 expression and upregulates claudin‐4 expression in HaCaT epidermal cells.^34^ Moreover, RA treatment reduces the cohesiveness of the stratum corneum, damages the skin barrier, and increases trans epidermal water loss by over‐expressing aquaporin 3 (AQP3) in epidermis, resulting skin dryness and sensitivity.^10^, ^36^

### 1.3 Solutions for tackling the retinoids instability and skin irritation

Researchers have attempted to improve the chemical stability and lessen the retinoid‐induced skin irritation through various strategies. These include:

1. Taking certain precautions during pack filling to control light and oxygen exposure. For example, airless and opaque packings.
2. Structural modifications of retinoids to increase their stability and reduce irritation.
3. Novel delivery systems by microencapsulate.
4. Association with other vitamins or soothing substances.

Therefore, our review aims to address the issues encountered during the topical cosmetic use of retinoid‐based formulations and provide current perspectives on better solutions for their wider application.

### 1.4 Structural modifications by retinoid derivatives

One of the biggest aspects to increase the chemical stability of retinoids is through structural modifications (Table 1). Semi‐synthetic and synthetic retinoid derivatives (including ‘atypical retinoids’) have been adopted to overcome the toxicity and instability of natural retinoids, and therefore serve as strong candidates for topical use in cosmetic industry. Due to the vulnerability of retinol to various factors such as light, oxygen, heat, lipid peroxidation and water, it is essential for retinol derivatives to possess not only anti‐aging properties but also low irritation and high stability.^37^

**TABLE 1:** Differentiation and functions of retinoids and retinols derivatives.

Name	Functions/advantages	Structures	Features of structural modifications
Hydroxypinacolone retinoate (HPR)	Directly bind to RXRs in the body without breaking down into RA form. Small molecular weight and does not require biotransformation	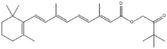	The combination of RA and pinacol, obtained from the modification of the active group of RA.^38^
N‐retinoyl‐D‐glucosamine (GRA)	Able to repair photoaged skin and does not cause transactivation activity of RAR with minimal skin irritation^39^	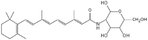	GRA was obtained by attaching glucosamine to the carboxyl group of ATRA.^39^
Retinyl retinoate	Greater inhibitory activity on c‐Jun to promote more collagen synthesis than retinol.^29^ Stimulate hyaluronic acid (HA) synthesis Lower intracellular toxicity and less induction of RAR activity	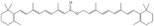	Formation of ester bonds between retinol and RA by condensation reactions.^40^
Bis‐retinamido methyl pentane (RS‐2A)	RS‐2A enhances the specific selectivity of RARs and reduces irritation and cytotoxicity; it also enhances its stability, bioactivity, and ability to stimulate more collagen synthesis^41^	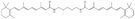	RS‐2A has two retinoid‐based portions at the ends of the molecule and a fatty chain in the middle, connected by an amide bond, a structural property that increases selectivity for retinoic acid receptors and enhances chemical stability and biological activity.^41^
Adapalene	Specifically recognize RAR‐β and RAR‐γ Increased stability against light exposure and oxidation.^42^ Anti‐inflammatory activities towards microcomedones.^43^	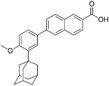	Modify with naphthoic acid derivative structure
Seletinoid G	Specifically binds RARγ Enhance wound healing and improve the skin barrier function in the dermis.^44^ Barely induced any skin irritation and erythema response compared to ATAR with increased type I collagen, elastin, and fibronectin‐1 in aged skin.^45^	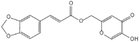	Converting 5‐hydroxy‐2‐(hydroxymethyl)‐4H‐pyran‐4‐one to the corresponding chloride which further reacted with potassium salt of 3,4‐(methylenedioxy) cinnamic acid.^44^, ^45^
Trifarotene	Regulate skin hydration via AQP3 and PADI1.^7^ Increased expression of RHCG and maintenance of the pH homeostasis of skin.^7^ Downregulate membrane MME to inhibit elastin fiber degradation and improve wrinkling.^46^ Trials that randomly assigned participants to evaluate the tolerability, safety, and effectiveness of trifarotene in treating acne have shown highly positive outcomes and minimal side effects.^47^	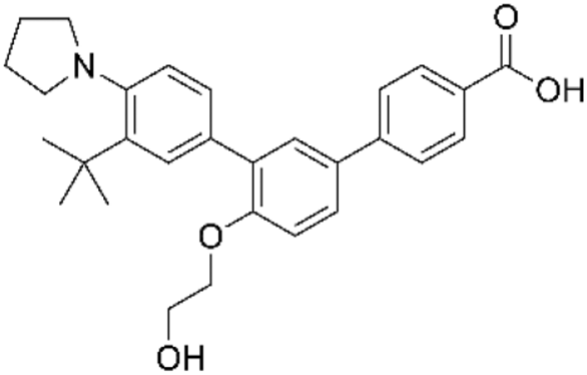	Structurally represents a terphenyl acid derivative and is a potent RAR‐γ agonist

HPR is formed by synthetic esther of 9‐cis‐RA. It is capable of directly binding to retinoid X receptor (RXRs) in vivo without requiring specific breakdown into the RA form through biological pathways.^25^ Study has shown that HPR significantly increased pro‐collagen production in skin models, induced less inflammatory cytokine interleukin‐1 alpha (IL‐1a) and retained high stability than ATRA.^48^ The combination of HPR and retinyl pro‐pionate (RP) further provide synergistic effect on tackling skin aging.^49^ In terms of in vivo study, coadministration of HPR and RP significantly enhanced fibroblast proliferation and promoted the gene expressions of type IV collagen, cellular retinol‐binding protein I (CRBP‐I), and RAR‐β. Clinical research also demonstrated that the combination receipt provided anti‐wrinkle and skin elasticity improvement with less adverse effects compared to retinoid under the same concentration.^49^ Meanwhile, encapsulating HPR and PR in the nanoparticles showed better stability and skin penetration effect than free HPR/PR combination, providing a better delivery modification for topical cosmetic use.^38^

A synthetic retinoid named as N‐retinyl‐D‐glucosamine (GRA) derived by adding glucosamine to the polar terminal group of ATRA. GRA, unlike RA, significantly reduced the expression of collagenase and did not induce skin irritation effects, presumably by the conversion of the toxic free carboxylic acid in the polar end group of RA into glucosamine at that site.^39^

In 2008, Kim et. al. had synthesized a novel hybrid of retinoid derivative: retinyl retinoate, from condensation reaction forming an ester bond between retinol and RA to enhance the thermal and photo‐stability.^29^ It also has lower skin irritation than RA, due to lower intracellular toxicity and less induction of RAR activity. Retinyl retinoate exhibits greater inhibitory activity on c‐Jun to promote collagen synthesis more effectively compared to retinol.^29^ Additionally, it has the ability to stimulate the synthesis of hyaluronic acid (HA).^50^ This group later also derived another synthesized retinoid derivative known as bis‐retinamido methylpentane (RS‐2A), with an amide bond connecting two retinyl moieties at both ends and an aliphatic chain in the middle.^41^ The structural changes in RS‐2A both increase the stability to oxidative degradation and enhance specific selec‐tivity to RARs, which further lead to reduce irritation and cytotoxicity. Especially, RS‐2A treatment in human dermal fibroblast did not promote the production of pro‐inflammatory cytokines MCP‐1 and IL‐8, thereby exhibited no retinoid‐induced skin irritation.^41^ Clinical data also support the application of retinyl retinoate and RS‐2A as additives in anti‐photoaging cosmetics.^40^, ^41^ However, more significant evidence with larger sample sizes should be provided to prove its potential benefits.^40^, ^41^, ^50^

Owing to the theory that non‐specific binding of retinoids to receptors in the body is a major cause of their irritation, there has been an emerging approach in the design of retinoid substances and their derivatives to specifically interact with certain RAR/RXRs.^51^ Third generation of retinoids such as adapalene specifically recognize RAR‐β and RAR‐γ via the adamantane substituent have been developed. Being a derivative of naphthoic acid, adapalene confers increased stability against light exposure and oxidation.^42^ It also exerts the anti‐inflammatory effects towards microcomedones by blocking the lipo‐oxygenase pathway and reducing neutrophil chemotaxis within the follicular units.^43^ Recently, researchers have designed the fourth generation synthetic retinoid using computer‐assisted molecular modeling: Seletinoid G.^45^ It structurally retains discriminative affinity towards RAR‐γ by transforming 5‐hydroxy‐2‐(hydroxymethyl)‐4H‐pyran‐4‐one to the corresponding chloride which further reacted with potassium salt of 3,4‐(methylenedioxy) cinnamic acid.^45^ Seletinoid G enhances wound healing by promoting the migration of keratinocytes and improves the function of the skin barrier by correcting abnormal collagen buildup in the dermis.^44^ Clinically, it barely induced any skin irritation and erythema response compared to ATRA and increased the expression of type I collagen, elastin, and fibronectin‐1 in aged skin.^45^

Another newly introduced fourth‐generation retinoid with selective action towards RAR‐γ is trifarotene. It structurally represents a terphenyl acid derivative and is a potent RAR‐γ agonist. Especially, trifarotene is able to regulate skin hydration via AQP3 and peptidyl arginine deiminase 1 (PADI1) to improve cutaneous barrier function. The increased expression of Rh family C glycoprotein (RHCG), which is responsible for transporting ammonium, indicates that trifarotene may affect the balance of NH4^+^/NH3 and, consequently, the maintenance of the pH homeostasis of skin.^7^ Moreover, trifarotene is able to downregulate membrane metalloendopep‐tidase (MME) to inhibit elastin fiber degradation and improve wrinkling or sagging during skin aging.^46^ Trials that randomly assigned participants to evaluate the tolerability, safety, and effectiveness of trifarotene in treating acne have shown highly positive outcomes and minimal side effects.^47^ As a result, trifarotene was granted approval by the FDA in 2019 for topical treatment of acne vulgaris, become the first retinoid to receive approval for the management of acne in the past two decades.^52^

### 1.5 Lipid‐based nano‐vehicles delivery system to increase the retinoid instability

Recent development in the nano‐encapsulation has opted the retinoid delivery system in terms of enhancing the bioavailability and safety. Nowadays, various delivery formulations have been developed including polymeric scaffolds (micro‐/nanofibers and hydrogels), nanoparticles and microparticles etc.^8^ One of the most efficient delivery systems is the lipid‐based carriers. Lipids can solubilize lipophilic compounds like retinoid and offer advantages in terms of biocompatibility, making them well‐suited for cosmetics applications.^53^ In this review, we mainly focus on the advances in lipid‐based nano‐vehicles facilitating the intrinsic instability and reducing the irritant effects of retinoids (Table 2; Figure 2).

**TABLE 2:** Different nano lipid‐based carriers and their features and functions for retinoids delivery.

Types of nano lipid‐based carriers	Characteristic features	Effective functions
Liposomes	Liposomes are spherical vesicles with a hydrophilic core surrounded by one or more phospholipid bilayers.	Enhance the transdermal penetration of retinoid substances, improve the bioavailability of retinol, and cause less irritation^54^, ^55^
Penetration‐enhancer vesicles (PEV)	Derived from deformable liposomes with phospholipids and penetration enhancers with hydrogen‐soluble diols	Temporarily reduce the barrier function of stratum corneum and enhancing the flexibility and movement of the vesicular bilayer^56^ Allow a gradual release of tretinoin, thus reducing its adverse effects^57^
Ethosomes	Derived from deformable liposome composed of phospholipids, ethanol and water	Facilitate deeper delivery of substances into ski Retinyl palmitate‐based ethosomal hydrogel improves tolerability with minimal local skin reactions^58^
Niosomes	Self‐assembly of nonionic amphiphiles in aqueous media, resulting in a closed bilayer structure.	Higher cutaneous retention^59^ Retard the drug photodegradation^60^ Enhance the cutaneous drug deposition when applied with penetration enhancer Labrasol®^61^ Suppress sebum secretion and hyperkeratosis without eliciting any skin irritation^62^
Nanoemulsions (NEs)	Heterogeneous dispersion of two immiscible liquids: water in oil (W/O) droplets or oil in water (O/W) droplets ranging from 20 to 200 nm and stabilize by an amphiphilic emulsifier	Low toxicity, non‐irritation, long‐term stability, and excellent wetting properties
Solid lipid nanoparticles (SLNs)	Exhibits a hydrophobic internal core composed of solid lipids and an external stabilizing layer formed by an amphiphilic surfactant	Improve the photostability and reduce the irritation^63^, ^64^ Combat the degradation of elastin and mitigate oxidative stress induced by UV exposure^64^, ^65^
Nanostructured lipid carriers (NLCs)	Incorporation of a liquid lipid, which forms a mixture of solid and liquid lipids within the nanoparticle structure.	Strong adhesion, enhanced skin penetration, and lubrication, which enhance the stability of the active ingredient^66^

**FIGURE 2:**
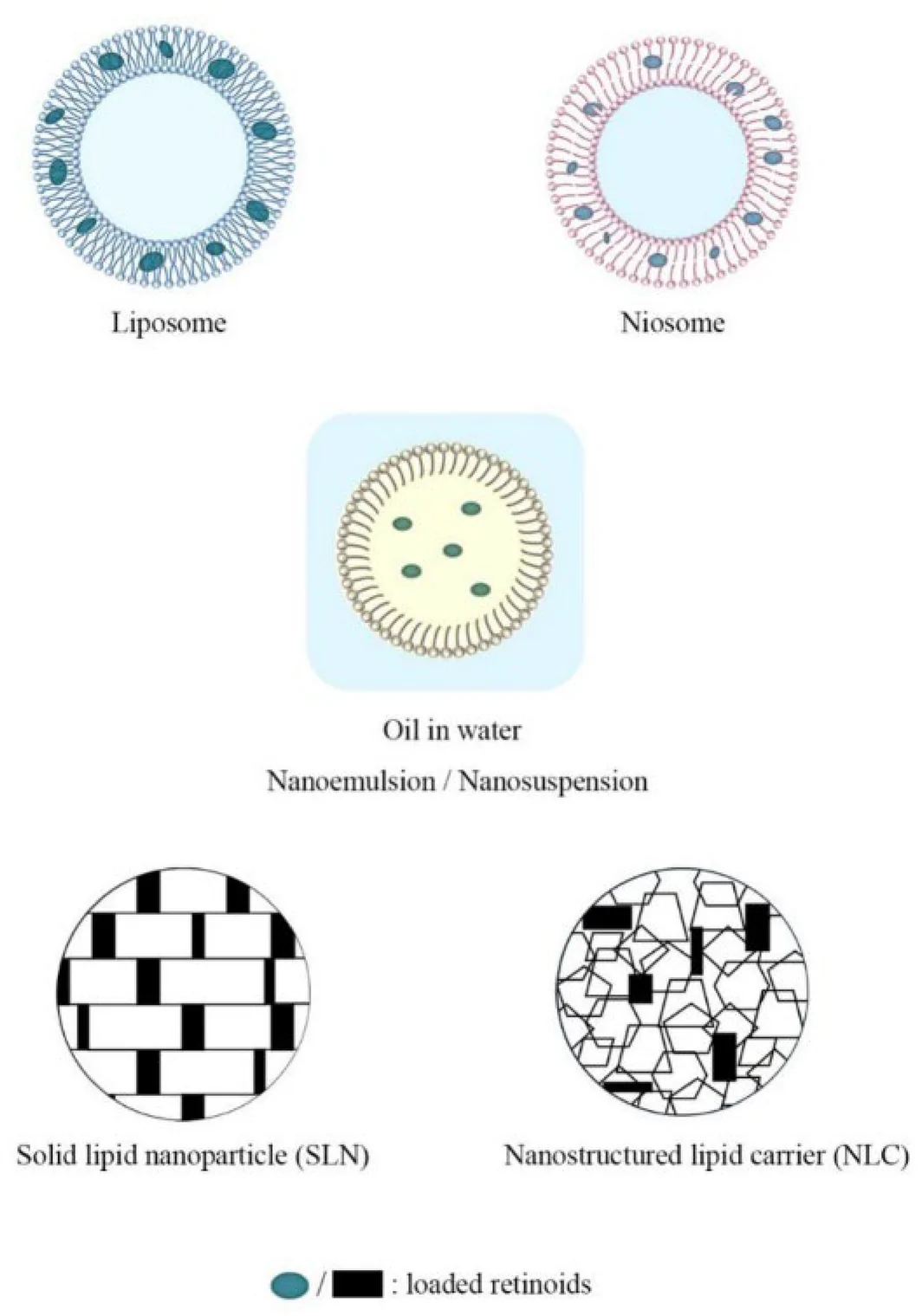
Schematic representation of nano lipid‐based carriers.

#### 1.5.1 Liposomes

Liposomes are microscopic structures ranging from 0.02 to 10 μm that are able to encapsulate lipophilic and hydrophilic compounds.^53^, ^67^ They consist of one or more concentric spheres of lipid bilayers with internal water compartments composed of membrane‐forming amphiphilic phospholipids or sphingolipids. Often, other bilayer components, such as cholesterol, are present in liposomes to enhance in vivo properties.^68^

Compared to conventional creams and gels, liposomes enhance the transdermal penetration of retinoid substances, increase the retinol content in the epidermis and dermis, improve the bioavailability of retinol, and cause less irritation.^55^, ^63^ The liposomes effectively protect retinoids from photo‐oxidation and improve their photostability.^54^ By incorporation of phytosterols, such as cholesterol (CH) and β‐sitosterol (SI) with liposome, Lee et al. have indicated their efficiency in maintaining the stability of retinol under 4°C and 25°C, with and without light, respectively.^69^ Nevertheless, the presence of double bond at C‐22 and C‐23 in stigmasterol (ST), another phytosterol with similar structure to SI, limited the interaction of liposome and sterol during retinol protection.^69^ Further system has been developed to construct the liposome‐sterol‐protein vehicle to facilitate the retinoids encapsulation. The use of liposomes/β‐lactoglobulin complex for retinoid delivery showed efficient bioavailability in the in vivo mice model.^70^ Therefore, liposome‐based retinoids have emerged as a promising approach for the treatment and prevention of skin aging.

Recent advancements in modifying vesicle compositions have been undertaken to create systems capable of transporting low molecular weight drugs and macromolecules to deeper tissues. Deformable liposomes or vesicles (UDV) are composed of phospholipids and an edge activator, typically a surfactant with a single chain to disrupt the lipid bilayers of the vesicles, leading to increased flexibility and deformability.^71^ One study found that the use of Tween 20‐based deformable liposomes significantly increased the skin permeability of retinol compared to the use of conventional neutral or negatively charged liposomes.^72^ The incorporation of a poorly water‐soluble retinoid into the lipid bilayer of liposomes is often constrained by the compound‐to‐lipid mass ratio. To overcome this limitation and address issues related to chemical stability, an alternative approach involves encapsulating water‐soluble retinoid/cyclodextrin (CyD) inclusion complexes within the aqueous phase of liposomes.^73^ However, a study comparing the physicochemical parameters of Tretinoin, a trans‐isomeric form of RA, revealed that the formulation of Tretinoin‐CyD‐UDV exhibited less chemical stability compared to Tretinoin‐UDV with a 20% lipid concentration, indicating the limited advantage of CyD deformable liposomes.^71^

Moreover, a novel family of deformable liposomes known as penetration‐enhancer vesicles (PEV) was developed to enhance trans‐dermal drug delivery. PEVs are made up of phospholipids and penetration enhancers with hydrogen‐soluble diols, which enable them temporarily reducing the barrier function of stratum corneum and enhancing the flexibility and movement of the vesicular bilayer.^56^ Bavarsad et al. indicated that PEVs allow a gradual release of tretinoin, thus reducing its adverse effects such as erythema, desquamation, burning, and increasing patient compliance.^57^

Ethosomes are another type of UDVs composed of phospholipids, ethanol and water to facilitate deeper delivery of substances into skin. RP‐based ethosomal hydrogel was proven to improve tolerability with minimal local skin reactions than conventional tretinoin formulation during acne treatments.^58^

#### 1.5.2 Niosomes

Nonionic surfactant‐based vesicles (niosomes) are formed by the self‐assembly of nonionic amphiphiles in aqueous media, resulting in a closed bilayer structure. Niosome as the carrier retarded the ATRA photodegradation.^60^ The use of niosomes to encapsulate tretinoin also demonstrated higher cutaneous retention compared to conventional liposomes or commercial formulation.^59^ Innovative niosomes that made by diolein, a double chain surfactant, showed enhancement in cutaneous tretinoin deposition when applied with penetration enhancer Labrasol® during in vitro percutaneous experiments.^61^ Recent study incorporating retinaldehyde‐loaded niosome also suggested its efficacy and safety in treating the acne by effectively suppressing sebum secretion and hyperkeratosis without eliciting any skin irritation.^62^

#### 1.5.3 Nanoemulsions

Different from the previously mention liposome and niosomes, which belongs to vesicular system, nanoemulsions (NEs) are heterogeneous dispersion of two immiscible liquids: water in oil (W/O) droplets or oil in water (O/W) droplets ranging from 20 to 200 nm and stabilize by an amphiphilic emulsifier.^53^ NEs have several advantages, including low toxicity, non‐irritation, long‐term stability, and excellent wetting properties. They are suitable for various formulation systems such as creams, sprays, and emulsions, as well as different routes of administration, particularly in dermal and transdermal drug delivery, to enhance the drug bioavailability.^74^ NEs with retinyl palmitate offered faster and deeper skin penetration, but induced loss skin flexibility during histological test.^63^ Study incorporated hydrophilic silica into the NEs improved the stability of the emulsions themselves while maintaining the photostability and long‐term storage stability of the retinol in the emulsions.^75^ Afornali et al. also showed that when retinoids were combined with NEs, there was an enhanced anti‐inflammatory response to tretinoin in ex vivo skin compared to other retinoids.^76^ Furthermore, NEs containing retinol microcapsules promoted biological processes related to protection against oxidative stress reactions, lipid metabolism in the extracellular matrix (ECM), and cell proliferation compared to commercial product from which tretinoin or microencapsulated retinol separately.^76^

#### 1.5.4 Solid lipid nanoparticles (SLNs) and nanostructure lipid carrier (NLCs)

SLNs and NLCs are lipid particle systems composed of solid lipids, or a combination of solid and liquid lipids, along with emulsifier.^53^ SLNs are the first‐generation lipoidal carriers that came into inception in the early 1990s as alternatives to liposomes, emulsions, and polymeric nanoparticles. At room temperature, SLNs exhibits a hydrophobic internal core composed of solid lipids and an external stabilizing layer formed by an amphiphilic surfactant (sometimes a co‐surfactant). They can be produced by simple and economical process and suitable to protect the drug from degradation and enable controlled release.^53^

Incorporating RA into SLNs led to a notable improvement in photostability and a considerable relief in irritation compared to other RA formulations.^63^, ^64^ Additionally, it effectively combats the degradation of elastin and mitigates oxidative stress induced by UV exposure.^64^, ^65^ SLN‐coated retinoid substances not only improve their photostability but also their epidermal permeability.^66^ The addition of chitosan to SLNs, followed by encapsulating tretinoin, has been discovered to enhance the encapsulation efficiency of SLNs.^77^ This formulation exhibits excellent physical stability, low cytotoxicity, and strong antibacterial activity against Acinetobacter pseudomonas and Staphylococcus aureus, and thus became a promising material for encapsulating RA.^77^

Although SLN systems offer numerous advantages, they do have some limitations: drug efflux and inadequate total payload.^77^, ^78^ To overcome the limitations of SLNs, a second generation of lipid nanoparticles called NLCs was developed. One key innovation of NLCs is the incorporation of a liquid lipid, which forms a mixture of solid and liquid lipids within the nanoparticle structure to disrupts the formation of ideal lipid crystals and reduce drug efflux. NLCs possess desirable characteristics such as strong adhesion, enhanced skin penetration, and lubrication, which enhance the stability of the active ingredient.^66^ Recently, Jun et al. developed the retinol‐loaded vacuum‐assisted lipid nanocarriers (VLN‐ROL) with particle size below 200 nm and exhibited strong negative surface charge of approximately −50 mV to prevent the VLN aggregation.^79^ The VLN‐ROL formulation demonstrated excellent stability with a significantly higher total amount of retinol delivered into the skin compared to the same concentration of bare retinol. In a 3D human skin model, the application of VLN‐ROL also led to an increase in epidermal thickness, higher relative percentage of dermal collagen area and reduced secretion of IL‐1α compared to the control and retinol alone, respectively.^79^ These results suggest that the VLN‐ROL has the potential to be an exceptional material for cosmetic and biomedical applications.

### 1.6 Retinoids combination with other substances

At present, there is a requirement to enhance the utilization of retinoids by not only enhancing their bioavailability through the utilization of RA derivatives and delivery systems but also by combining them with other substances to improve stability and minimize their irritant properties (Table 3).

**TABLE 3:** Retinol combinations and their effects after combinations.

Name		Main characteristics	Effects after combinations
	Niacinamide (vitamin B3)	Niacinamide increases skin elasticity to improve wrinkles and prevent the transfer of melanin cells to keratin‐forming cells to achieve a whitening effect	The combination attenuates AQP3 expression to reduce moisture loss from the skin and alleviate the side effects of retinol‐induced dryness; thickens the stratum corneum and reduces skin irritation^80^
Vitamins	Vitamin C	Vitamin C is an antioxidant, inhibits tyrosinase activity, and promotes collagen production	The combination stimulates ceramide synthesis, promotes regeneration of skin surface lipids, increases collagen synthesis, improves skin aging, and relieves dry skin caused by retinol^81^, ^82^
	Vitamin E	Vitamin E has antioxidant, anti‐inflammatory, and skin smoothing ability to increase moisture in the stratum corneum	The combination significantly reduces skin lipid oxidation and skin inflammation^83^
Soothing and hydrating agents	Hyaluronic acid (HA)	HA maintains the skin's moisture and extracellular space	The combination has free radical scavenging activity, slows down the inflammatory response, and reduces irritation^84^
	Alpha‐hydroxy acids (AHAs)	AHAs have moisturizing and exfoliating properties	Double‐conjugated of retinol/retinoid and AHA improved skin clarity and pores with transient adverse effects observed^85^
	Aloe vera	Aloe vera has anti‐inflammatory, anti‐irritant, wound‐healing and antibacterial properties	The combination relieves skin redness and irritation associated with retinol^86^
	Water soluble cannabidiol (CBD)	CBD has resistance to photodamage, anti‐inflammatory effect, and moisturizing ability	The combination protects the skin through antioxidant and anti‐inflammatory mechanisms and reduces the irritation of retinol^87^
Antioxidants and metal chelator	[S,S]‐EDDS and pentaerythritol 3,5‐di‐tert‐butyl‐4‐hydroxyhydrocinnamate (PBHC)	Stabilize the easily degradable retinol and replace non‐environmentally‐friendly EDTA and BHT	The combination prevents retinol against degradation to allow long‐term storage and further enhanced the skin absorption of retinol^49^

#### 1.6.1 Vitamins

To tackle the side effect induced by RA treatments, studies have demonstrated that certain vitamins can effectively improve the skin barrier function of individuals with atopic dry skin. Niacinamide, also referred to as nicotinamide, is a variant of vitamin B3 that exists in human cells as a precursor to nicotinamide adenine dinucleotide (NAD^+^).^88^ Niacinamide, when used topically, has been adopted as moisturizing and soothing ingredient to reduce wrinkles and freckles, improve skin elasticity, and inhibit the transfer of melanocytes to keratinocytes.^89^ Moreover, when combing with retinol, niacinamide reduces the expression of AQP3 induced by retinol via inhibiting the epidermal growth factor receptor/extracellular‐signal regulated kinase (EGFR/ERK) signaling pathway.^80^ This ultimately leads to an improvement of water permeability and retention, which can help alleviate the side effects of retinol‐induced dryness. Clinical studies have indicated that the combination of nicotinamide and retinol results in a notable reduction in the expression of matrix metalloproteinases (MMP‐1, MMP‐2, MMP‐9, MMP‐14), IL‐6, MCP‐1, and Macrophage migration inhibitory factor (MIF), while increasing the expression of transforming growth factor beta (TGF‐β) compared to using retinol alone.^90^ Additionally, introducing nicotinamide in high‐concentration retinoids has been shown to thicken the stratum corneum, leading to significantly less skin irritation and improved patient tolerance.^91^

Vitamin C, which can be directly absorbed by the skin, exhibits antioxidant properties, acts as an anti‐free radical agent, inhibits the production of tyrosinase, and contributes to reducing melanin levels in human.^81^, ^92^ It acts as a cofactor for proline and lysine hydroxylase enzymes, which play a crucial role in the stability and formation of collagen fibers, making vitamin C as an excellent ingredient for both skin whitening and anti‐aging.^93^ The combination of vitamin C and retinoid substances can promote the synthesis of ceramides and the regeneration of skin surface lipids, thereby stimulating sebum secretion on the face and neck. This, in turn, enhances the barrier function and alleviates dry skin commonly associated with the use of retinoids.^82^, ^94^, ^95^ Moreover, clinical trials of a dual product regimen with 0.5% retinol and 30% vitamin C significantly improve wrinkles, hyperpigmentation, skin firmness and smoothness upon a 12‐week treatment.^96^

Vitamin E, another well‐known antioxidant, is commonly utilized for shielding the skin against atopic dermatitis.^97^ Due to its directly reactive oxygen species (ROS) neutralization capacity and documented anti‐inflammatory properties, vitamin E is a highly effective choice for topical application, providing enhanced protection against retinoid‐induced inflammation and irritation.^83^ When combined with retinoid substances, vitamin E demonstrates a significant reduction in the oxidation of skin lipids and can effectively decrease the expression of TNF‐α, thereby relieving the inflammatory response.^83^

#### 1.6.2 Soothing and hydrating agents

HA is a prominent non‐sulfated glycosaminoglycan (GAG) found in the skin. This molecule is widely recognized for its exceptional water‐retaining ability, which contributes to skin hydration and leads to improved firmness, elasticity and maintains the moisture and extracellular space of the skin. Coadministration of hydrating ingredient HA and 0.05% tretinoin significantly minimized irritation and improved patient tolerance.^98^ In recent studies, scientists have combined HA with ATRA to create a novel amphiphilic compound called HA‐ATRA.^84^ This compound effectively activates retinoid signaling, demonstrates noteworthy free radical scavenging properties, reduces the expression of pro‐inflammatory factors such as IL‐6 and IL‐8, and exhibits favorable long‐term stability.^84^ However, further studies need to be conducted to evaluate the pros and cons over other retinoids for this amphiphilic compound.

Alpha‐hydroxy acids (AHA) are non‐toxic organic acids (such as glycolic, lactic, and malic acids) with one or more hydroxyl groups attached to the alpha position of the acid. They have been reported to have moisturizing and exfoliating properties as pH adjusters and humectants in cosmetic formulations.^99^ The combinatory effects of 30–35 mM lactic acid and 10–15 mg/mL retinol towards dermal fibroblasts showed better maintaining of elastin after UV exposure.^100^ Bioengineered ethyl lactyl retinoate, an AHA retinoid conjugate, was developed to deliver AHA and retinoids in a hydrolysis‐based time‐released fashion and minimized retinoid‐induced irritation like dryness and flaking.^101^ Clinically, the treatment of 12‐week double‐conjugated of retinol and AHA to blemish‐prone skin demonstrated improvements skin clarity and pores with transient adverse effects observed.^85^ Another clinical study of double conjugation of the retinoid with AHA and ethanol for 8‐week significantly reduce the inflammatory and noninflammatory lesion in acne patients.^102^

For numerous years, plant extracts have been utilized in both medicinal and cosmetic applications. Aloe vera, found in gels, possesses beneficial properties such as anti‐inflammatory, anti‐irritant, wound healing, and antibacterial effects. When combined with RA, aloe vera exhibits synergistic effects in reducing inflammation and promoting wound healing. Additionally, aloe vera helps alleviate skin redness and irritation. The combination of aloe vera and retinoids has shown good tolerability.^86^ Another plant extract water soluble cannabidiol (CBD) was found to decrease inflammation during skin diseases.^103^ The antioxidant and anti‐inflammatory activity of CBD is regulated via NF‐E2–related factor 2 (Nrf2)‐target genes, including *Heme Oxygenase 1* (*HMOX1*) but via a mechanism independent of Nrf2.^104^ On the other hand, retinol tends to suppress the expression of Nrf2‐target antioxidative genes.^105^ By incorporating CBD, there is a potential to restore the antioxidative effects that may be inhibited by retinol, while still retaining the skin‐strengthening properties. Retinol combined with water soluble CBD can protect the skin through antioxidant and anti‐inflammatory mechanisms and reduces retinol irritation, thereby improving efficacy and tolerability.^87^

#### 1.6.3 Antioxidants and metal chelator

Conventionally, retinoids are stabilized through the use of specific additives, including antioxidants called butylated hydroxytoluene (BHT) or butylated hydroxyanisole.^106^ Additionally, metal chelator such as ethylenediaminetetraacetic acid (EDTA) and its salts are commonly employed in combination. The applications of these formulations have been highly valued in cosmetic formulations by preventing thermal isomerization and decomposition of all‐trans‐retinol caused by lipid peroxides.^75^ However, BHT and the complexes formed by EDTA and metals have raised concerns due to its sub‐optimal environmental profile and associated with environmental issues and controversies.^106^, ^107^ Therefore, there is an urgent need to develop a new system that offers eco‐friendly alternatives while maintaining the stability of retinoids.

Recently, Wang et. al. developed an environmentally friendly system to stabilize the easily degradable retinol.^49^ They adopted Ethylenediamine‐ N,N′‐disuccinic acid ([S,S]‐EDDS) to replace EDTA and pentaerythritol 3,5‐di‐tert‐butyl‐4‐hydroxyhydrocinnamate (PBHC) to replace BHT in retinol formulations. This new combination prevented retinol against degradation to allow long‐term storage and further enhanced the skin absorption of retinol.^49^ Thus, this innovative stabilization system represents a significant step towards the development of sustainable solutions for stabilizing retinol.

## 2 FUTURE DIRECTION AND CONCLUSION

Through advancements in formulation techniques, the chemical instability of retinoids has been mitigated, ensuring their efficacy and potency over extended periods. Moreover, efforts have been made to minimize skin irritation associated with retinoids use, such as structure modification of retinoid derivatives, development of novel delivery systems and the incorporation of soothing ingredients. However, future perspectives in this field are crucial for further advancements.

Recently, the Scientific Committee for Consumer Safety (SCCS) in the European Union issued a new regulation imposing prohibitions and future restrictions on the use of retinol in commonly used skincare products.^108^ This includes retinol, retinyl acetate, and retinyl palmitate. The SCCS has considered the combined exposure of vitamin A from both cosmetics and diet, and has precisely evaluated the safety exposure value concentration as 0.05% retinol equivalent (RE) for body lotion and 0.3% RE for other leave‐on and rinse‐off products.^108^ Based on the SCCS evaluation, it can be inferred that the use of Vitamin A in cosmetic products beyond specific concentrations poses potential health risks to humans. Following a thorough assessment, the SCCS stated that a maximum contribution of 150 μg RE/day (equivalent to 10% of the tolerable upper intake level) from cosmetic products could be considered acceptable. This value applies to various groups, including women of childbearing age, middle‐aged women, elderly women with declining bone density, as well as men and children above the age of 10. Such regulation could significantly influence the formulation of cosmetic products containing retinol and its equivalents not only within the European Union but also globally. For risk management issue, it is also necessary to provide a notice to consumers who are already exposed to Vitamin A, cautioning them about the potential for excessive intake through dietary sources and supplements when using products containing Vitamin A derivatives.

Clinical research with novel delivery system focused on enhancing the stability of retinoids and reducing skin irritation should be investigated for larger patient cohorts and longer term to ensure the promising effects. In future studies, the inclusion of placebo control and blinding techniques for both the patient and investigator will provide valuable insights into the effects of topical retinoids. In terms of molecular research, the development of in vitro three dimensions (3D) models, such as 3D skin model and skin organoids has offered alternatives to recapitulate the cellular physiology than ordinary cell line cultures. Cellular studies adopting these models, in conjunction with ultrasensitive high‐throughput analytical tools, offer valuable insights into the molecular changes associated with retinoid‐induced irritation. By employing advanced analytical techniques, researchers can decipher intricate molecular alterations occurring in the skin upon retinoids application. This comprehensive understanding of the underlying mechanisms provides crucial implications for identifying key pathways and targets involved in irritation. Moreover, it enables the development of targeted strategies to mitigate or prevent these adverse effects. The combination of molecular studies and advanced analytical tools holds immense potential in unraveling the complexities of retinoid‐induced irritation, paving the way for the development of safer and more effective retinoids formulations in the future.

## AUTHOR CONTRIBUTIONS

*Conceptualization*: P.S. and Z.Y.D. *Research search literature methodology*: J.M.Z and N.Z. *Writing—original draft preparation*: J.M.Z and N.Z. *Writing—review and editing*: J.M.Z, N.Z. Q.L.S. and P.S. *Figure generation/visualization*: J.M.Z and N.Z. All authors have read and agreed to the published version of the manuscript.

## CONFLICT OF INTEREST STATEMENT

The authors declare that the research was conducted in the absence of any commercial or financial relationships that could be construed as a potential conflict of interest.

## ETHICS STATEMENT

An ethics statement is not applicable because this study is based exclusively on published literature.

## Data Availability

Data sharing not applicable to this article as no datasets were generated or analysed during the current study.
